# Cadmium Removal from Synthetic Waste-Water Using TiO_2_-Modified Polymeric Membrane Through Electrochemical Separation System

**DOI:** 10.3390/polym18020150

**Published:** 2026-01-06

**Authors:** Simona Căprărescu, Roxana Gabriela Zgârian, Grațiela Teodora Tihan, Alexandru Mihai Grumezescu, Eugenia Eftimie Totu, Daniel Costinel Petre, Cristina Modrogan

**Affiliations:** 1 Department of Inorganic Chemistry, Physical Chemistry and Electrochemistry, Faculty of Chemical Engineering and Biotechnologies, National University of Science and Technology POLITEHNICA Bucharest, 1-7 Gheorghe Polizu Street, 011061 Bucharest, Romania; simona.caprarescu@upb.ro; 2 Department of General Chemistry, Faculty of Chemical Engineering and Biotechnologies, National University of Science and Technology POLITEHNICA Bucharest, 1-7 Gheorghe Polizu Street, 011061 Bucharest, Romania; roxana.zgarian@upb.ro; 3 Department of Science and Engineering of Oxide Materials and Nanomaterials, Faculty of Chemical Engineering and Biotechnologies, National University of Science and Technology POLITEHNICA Bucharest, 1-7 Gheorghe Polizu Street, 011061 Bucharest, Romania; agrumezescu@upb.ro; 4 Research Institute of the University of Bucharest, University of Bucharest, 90 Panduri Street, 050663 Bucharest, Romania; 5 Department of Analytical Chemistry and Environmental Engineering, Faculty of Chemical Engineering and Biotechnologies, National University of Science and Technology POLITEHNICA Bucharest, 1-7 Gheorghe Polizu Street, 011061 Bucharest, Romania; eugenia.totu@upb.ro (E.E.T.); danielpetre.empresa@hotmail.com (D.C.P.); cristina.modrogan@upb.ro (C.M.)

**Keywords:** modified membranes, TiO_2_ nanoparticles, electrochemical separation system, cadmium removal, structural characterization, impedance spectroscopy

## Abstract

In this paper, a new polymeric membrane including polymers (cellulose acetate, polyethylene glycol 400), copolymer poly(4-vinylpyridine)-block-polystyrene, and TiO_2_ nanoparticles were synthesized by the phase inversion method. In order to investigate the presence and the influence of the TiO_2_ nanoparticles on the membrane matrix, a polymeric membrane without TiO_2_ nanoparticles was prepared by the same preparation method. The structure of the polymeric membranes was characterized by several techniques, such as Fourier transform infrared spectroscopy and scanning electron microscopy coupled with energy-dispersive X-ray spectroscopy, thermogravimetric analysis, and impedance spectroscopy. Also, the water contact angle, water retention, and porosity were determined. The results showed that the TiO_2_ nanoparticles were incorporated into the pores and onto the surface of the polymeric membrane, which resulted in a more uniform structure. In addition, these polymeric membranes were tested for the removal of cadmium ions from synthetic waste-water using a laboratory-scale electrochemical separation system with a custom-built setup. The results showed that the polymeric membrane with TiO_2_ nanoparticles showed a high cadmium ions removal rate (95.53%), compared to the polymeric membrane without TiO_2_ nanoparticles (85.29%), after a 1.5 h electrochemical separation test. The final results indicated that the polymeric membranes prepared with TiO_2_ nanoparticles had excellent thermal stability and exhibited the best ionic conductivity. The electrochemical separation system proved that the obtained polymeric membranes effectively remove cadmium from the synthetic waste-water.

## 1. Introduction

Drinking water resources and freshwater needs are crucial for environmental safety and human health in many regions of the world due to rapid urbanization, industrialization, human activities, the increasing world population, and climate change.

Annually, various industrial processes (e.g., metal plating; battery manufacturing; iron and steel production; electroplating; mining; smelting; stabilizing plastics, alloys, cement, and pigments; fossil fuel combustion; high-phosphate fertilizers; municipal and sewage sludge incineration mining) discharge tons of waste materials containing multiple toxic heavy metals (e.g., chromium (Cr), nickel (Ni), lead (Pb), copper (Cu), zinc (Zn), cobalt (Co), mercury (Hg), cadmium (Cd), and iron (Fe)) into the environment [1,2]. The discharge of waste-waters containing heavy metals without proper treatment is a major problem for the environment, and also for the health of various life forms (e.g., animals, plants, humans, aquatic organisms, or microorganisms), because heavy metals cannot be biodegraded. Their presence in natural/fresh waters, soil, and groundwater leads to the deterioration of the ecosystems. Cadmium (Cd) is a highly toxic heavy metal that causes serious irreversible effects on both human health and the environment even in low concentrations, doses, and quantities. The World Health Organization (WHO) [3] has set the limit for Cd in drinking water at 0.003 mg L^−1^, and the United States Environmental Protection Agency (EPA) has set the maximum contaminant level for Cd at 5 μg L^−1^, which is the same in the European Union (UNEP) [4], and it has also recommended the maximum limit for Cd uptake in drinking water (0.5 mg kg^−1^ per day). Meanwhile, the Council Directive 98/83/EC regulating the quality of water intended for human consumption sets the maximum limit at 0.005 mg L^−1^ in humans [3,4,5,6], because Cd is carcinogenic, mutagenic, and consumption over that limit has severe effects on human health (e.g., kidneys and liver damage, lungs and skeletal malformations, central nervous system damage, endocrine tissues damage, elevated blood pressure and cellular functions, cardiovascular disorders, chronic pulmonary problems and several cancers) [1,2,7].

Over time, several conventional and modern methods or processes have been employed to remove the cadmium ions (Cd^2+^) from various waste-waters, such as incineration, adsorption, coagulation and flocculation, chemical oxidation, precipitation, electrolysis [8,9,10], evaporation, ion exchange, biological and photocatalytic methods, and advanced membrane separation processes [7,11,12,13]. Most of these methods or processes require chemicals, generate large quantities of secondary waste, and produce chemical sludge, which leads to increased operational, management, or processing costs. They also require significant space and infrastructure, making them less suitable for laboratory or industrial-scale treatment. The choice of method or process for treating waste-water containing metal ions depends on many factors, such as waste-water characteristics (e.g., composition, pH, conductivity), concentration or quantity of metallic ions, operating costs, maintenance costs, and their overall efficiency.

However, membrane separation processes (e.g., electrodialysis, nanofiltration, microfiltration, ultrafiltration, reverse osmosis, forward osmosis, and membrane distillation) have been applied widely in the field of water and waste-water treatment due to their small size, low energy consumption, and low initial cost [7,11,12,13,14]. Due to their economic feasibility combined with their low energy requirements and the ease with which membrane modules can be scaled up, these membrane separation processes are also being exploited in other industrial sectors such as biotechnology, nanotechnology, and membrane energy devices [15,16]. For these processes, various membranes containing polymers (e.g., cellulose acetate, chitin, polyethylene glycol, polyvinyl alcohol, poly(vinyl chloride), polyacrylonitrile, polysulfone, polyimide, polyaniline, polyethylene, polypropylen, poly(ethyleneimine), poly(vinylidene fluoride)) and nanoparticles (i.e., SiO_2_, TiO_2_, Al_2_O_3_, ZnO, MgO, SnO_2_, ZrO_2_) were developed and used for the treatment of water and waste-waters containing metallic ions (e.g., Cu^2+^, Ni^2+^, Zn^2+^, Pb^2+^, Fe^2+^, Cd^2+^) [12,13,14,17,18]. These membranes present many properties and advantages such as hydrophilicity, permeability and selectivity, processability, antifouling resistance, high porosity, chemical and thermal stability, mechanical strength, flexibility for surface modification, versatility, low cost factor, and environmental sustainability [14,17,18,19]. DeFriend et al. [13] found that the fabricated membrane containing alumina nanoparticles has a high porosity and a good permeability. Filimon et al. [14] determined that the mechanical properties, water resistance, and antifouling performance of polyvinyl alcohol-based membranes were improved by the inclusion of TiO_2_ nanoparticles in the polymeric membrane matrix. Ayyaru et al. [19] reported that the addition of graphene oxide–ZnO nanoparticles in a poly(vinylidene fluoride) membrane improved membrane porosity, wettability, water flux, and antifouling properties.

Electrodialysis (ED) is a stationary membrane separation process that uses the ion-selective membrane, semi-permeable membranes or ion-exchange membranes for separating pollutants under the action of an electric field. ED was first applied for water desalination; then, over time, it was applied for waste-water treatment from various industries (e.g., industrial laundry waste-water, food processing industries, cosmetics and pharmaceutical products, galvanic industries, mining and refining, agricultural water, paper industry) [20,21,22,23,24]. ED was applied for the treatment of different waste-waters containing various heavy metals (i.e., nickel, copper, zinc, lead, cadmium, iron, manganese) due to its advantages such as high ion removal efficiency, low sludge production, and excellent rejection and removal performance. ED also provides both technical and economic benefits, has easy installation and operation, and can be operated as continuous production or batch production [7,20,21,22,23,24]. The drawbacks of the electrodialysis process are its high energy consumption and high cost if used in seawater desalination; membrane fouling; the high initial costs (the initial cost of the membranes and infrastructure can be significant); that it requires a certain level of conductivity of the water to be treated; the lack of significant effect against biological pollutants; and the limitation of the membranes’ lifespan [24,25,26,27,28]. Lee [24] reported that the studies on the treatment of waste-waters contaminated by high concentrations of cadmium are very limited. Santarosa et al. [26] found that the electrodialysis process is a flexible process used in the treatment of effluents after galvanic phosphatization. Mikhaylin and Bazinet [29] reported that ED has problems, such as organic fouling, colloid fouling, and scaling caused by inorganic crystals. Căprărescu et al. [21] prepared polymeric membranes based on polyvinyl alcohol and SiO_2_ nanoparticles for the treatment of waste-waters containing zinc ions by the electrodialysis process. They found that the inclusion of SiO_2_ nanoparticles in the membrane matrix improved the resistance, proton conductivity, and removal efficiency of the polymeric membrane. Hosseini et al. [30] fabricated heterogeneous ion exchange membranes filled with Fe_3_O_4_ nanoparticles for Pb removal from waste-water. The results indicated that the higher potential and selectivity for Pb^2+^ were obtained for the membranes with higher concentrations of Fe_3_O_4_ nanoparticles (8%wt).

In this paper, the efficiency of a new type of polymeric membrane, with and without TiO_2_ nanoparticles (CA-PEG-PVST0 and CA-PEG-PVS0 membranes), for the removal of cadmium ions from synthetic waste-water was investigated using a laboratory-scale electrochemical separation system with a custom-built setup. The system works on the principle of the electrodialysis process. The novelty of this paper consists of the preparation of the possible perm-selective or ion-selective polymeric membranes and also the construction of a laboratory-scale electrochemical separation system. The polymeric membranes prepared by the phase inversion method were further analyzed by fourier transform infrared spectroscopy (FTIR), scanning electron microscopy (SEM) coupled with energy-dispersive X-ray spectroscopy (EDS), thermogravimetric analysis (TGA), and impedance spectroscopy to investigate the influence of the TiO_2_ nanoparticles added into the polymeric membrane matrix (surface hydrophilicity, wetting properties, chemical and thermal stability) and, also, to observe the interaction between cadmium ions and TiO_2_ nanoparticles. Also, the contact angle and porosity were determined. The electrochemical separation system performance and efficiency of the polymeric membranes were evaluated by determining the removal rate of Cd^2+^, the current efficiency, the mass flow of Cd^2+^, the specific energy consumption, and the mass transfer coefficient. The ionic conductivity of the polymeric membranes was also investigated before and after the electrochemical separation tests.

## 2. Materials

Cellulose acetate (CA) (powder), polyethylene glycol 400 (PEG) (liquid), poly(4-vinylpyridine)-block-polystyrene (PVS) (powder), sulfuric acid, and titanium dioxide (TiO_2_) (nanopowder, <100 nm particle size) were purchased from Sigma-Aldrich (Merck KGaA, Darmstadt, Germany). Glacial acetic acid and cadmium sulfate octahydrate (anhydrous salt) were provided by Chimpoar S.A. (Bucharest, Romania). All chemicals are analytical reagent grade and were used in the investigations as received.

## 3. Methods

### 3.1. Preparation of the Polymeric Membranes

**Figure 1:**
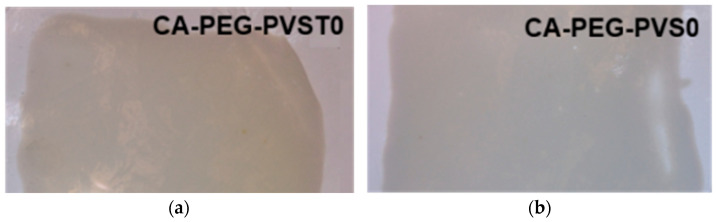
Images of the obtained polymeric membranes: (**a**) with TiO_2_ nanoparticles (CA-PEG-PVST0 membrane); (**b**) without TiO_2_ nanoparticles (CA-PEG-PVS0 membrane).

The polymeric membranes were obtained by the phase inversion method at room temperature (23 ± 1 °C). The homogeneous solution was prepared by dissolving CA (1 g) and PEG (6 cm^3^) with glacial acetic acid (25 cm^3^) by stirring for 30 min at 120 °C. The stirring was carried out at 350 rpm, using a magnetic stirrer with heating (DLAB MS-H380 Pro, DLAB SCIENTIFIC CO., Beijing, China). After that, the poly(4-vinylpyridine)-block-polystyrene (0.07 g) was added gradually to the homogeneous solution under continuous magnetic stirring (350 rpm) for 3 h. To the viscous and homogeneous solution obtained, TiO_2_ nanoparticles (0.08 g) were added under continuous magnetic stirring (350 rpm) for 2 h to completely dissolve all constituents. The resulting viscous and clear solution was cooled at room temperature (23 ± 1 °C) for 15 min to remove micro-bubbles. After that, the polymer solution obtained was poured and applied to a glass plate using a special knife with a thickness of 0.73 mm. The obtained polymer film was immediately immersed in a bath containing distilled water and left until it was completely detached from the glass plate. After 24 h, a smooth, uniform, homogeneous, compact, elastic, and white polymeric membrane containing TiO_2_ nanoparticles (CA-PEG-PVST0 membrane) was obtained. To indicate the presence of TiO_2_ nanoparticles in the polymer matrix, a polymeric membrane without these nanoparticles (CA-PEG-PVS0 membrane) was prepared, using the same preparation method. The obtained polymeric membranes are indicated in Figure 1.

The prepared polymeric membranes involved an intercalated network of polymeric materials, with and without TiO_2_ nanoparticles, in which CA, due to its functional properties, such as high chemical stability, high mechanical stability and high hydrophilicity, can constitute the basic matrix of the structure, while the use of PEG can improve the hydrophilicity and flexibility of the membrane. The poly(4-vinylpyridine)-block-polystyrene (copolymer) (poly(4-vinylpyridine) is a cationic polymer) can provide specific interactions with different ions, and the TiO_2_ nanoparticles can improve the structural stability and help with the removal of metallic ions from waste-water. Preliminary tests on the preparation of polymeric membranes have indicated that the incorporation of less than 0.08 g of TiO_2_ nanoparticles does not ensure good chemical and thermal stability of the membrane, not does it ensure high ionic conductivity or high efficiency in waste-water treatment. An amount greater than 0.08 g of TiO_2_ nanoparticles leads to an agglomeration of nanoparticles in the membrane structure, which affects their chemical stability. CA, PEG, poly(4-vinylpyridine)-block-polystyrene (copolymer), and the TiO_2_ nanoparticles were used for the preparation of a possible perm-selective or ion-selective polymeric membrane.

The thickness of each obtained polymeric membrane was around 0.4 mm. The measurements were performed using a high-precision electronic digital caliper with a digital display (Dasqua 2015-1005-A, Dasqua S.R.L., Cornegliano Laudense, Italy).

The obtained polymeric membranes (CA-PEG-PTVS0 and CA-PEG-PVS0 membranes) were tested in an electrochemical separation system to observe the effect on TiO_2_ nanoparticles in the polymeric membrane matrix and also to evaluate the efficiency of the polymeric membranes in removing cadmium ions from synthetic waste-water.

### 3.2. Electrochemical Separation System Construction and Setup

**Figure 2:**
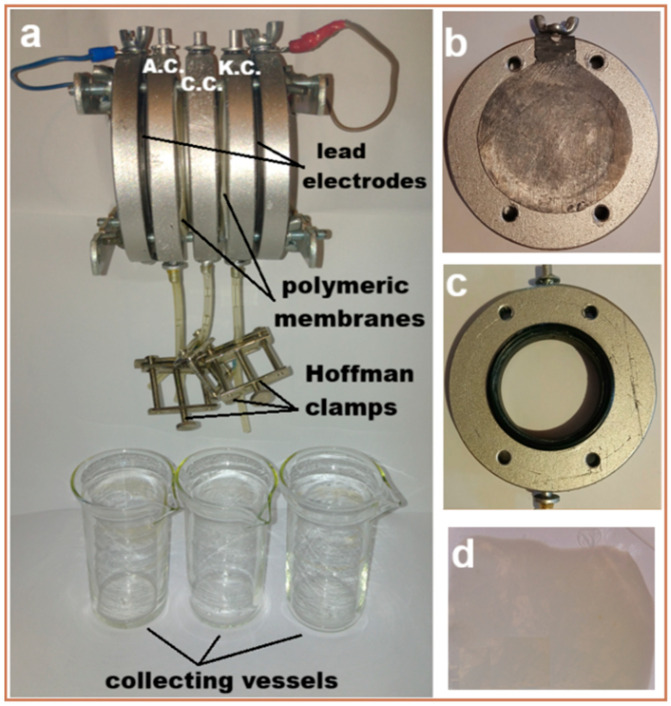
Images of the laboratory-scale electrochemical separation system with a custom-built setup: (**a**) elements of the electrochemical separation system; (**b**) lead electrode attached to the support; (**c**) circular compartment; and (**d**) polymeric membrane.

In order to evaluate the efficiency of the fabricated polymeric membranes (with and without TiO_2_ nanoparticles (CA-PEG-PVST0 and CA-PEG-PVS0 membranes)), a laboratory-scale electrochemical separation system with a custom-built setup was constructed from high-density polyethylene material (Figure 2). The electrochemical separation system consists of two parallel circular plate electrodes (made of lead, purity 99.9%; anode and cathode (on which the metallic ions were deposited)) placed at the ends of the electrochemical separation system (see Figure 2a,b), three circular compartments (anodic (concentrate solution) (A.C.), central (dilute solution) (C.C.), and cathodic (electrode rinse solution) (K.C.)) (see Figure 2a,c), and three glass vessels for collecting solutions containing cadmium ions. The anode lead is positioned on the left side of the electrochemical separation system, and the cathode lead is positioned on the right side of the electrochemical separation system (at the ends of the system; both electrodes are fixed to the support (see Figure 2a,b)). The two identical polymeric membranes were placed between the compartments (see Figure 2a,d). The thickness of each compartment was 9.85 mm. The effective surface area of each polymeric membrane and each active electrode in the electrochemical separation system was approximately 1896 mm^2^. The internal and external diameters of each compartment were 44.85 mm, and 89.11 mm, respectively. The thickness of each lead electrode was 0.31 mm. Lead electrodes are malleable, easy to clean and handle within the electrochemical separation system, and are both chemically and electrochemically inactive in contact with synthetic waste-water introduced into the electrochemical separation system; no other ions are removed during the electrochemical separation process. The electrochemical separation system was operated at a constant voltage (10 V) imposed on the electrodes by a high-precision DC-stabilized power supply (AXIOMET AX-3005D), at room temperature (23 ± 1 °C), for 1.5 h (each experiment). The compartments were opened at the top to reduce hydrogen accumulation in the electrochemical separation system.

The electrochemical separation tests were accomplished using an initial synthetic waste-water (1 L) prepared by cadmium sulfate octahydrate (CdSO_4_·8H_2_O) (anhydrous salt), sulfuric acid 1 N (50 mL), and distilled water to obtain a waste-water feed of 1500 ppm (1500 mg L^−1^) cadmium ions. All compartments were filled with a total volume of approximately 75 cm^3^ of feed synthetic waste-water containing cadmium ions (Cd^2+^). The initial synthetic waste-water and solutions collected after the electrochemical separation tests were analyzed using a UV-Vis spectrophotometer (SP-830+, Metertech Inc., Nankang, Taipei, Taiwan), at 226.5 nm wavelength, to determine the cadmium ions concentrations.

### 3.3. Membrane Characterization

#### 3.3.1. Morpho-Structural Investigation of the Polymeric Membranes

The Fourier transform infrared (FTIR) spectra for all polymeric membranes were recorded with a Perkin Elmer Spectrum 100 FTIR spectrophotometer (PerkinElmer, Ltd., London, UK). FTIR spectra were acquired in the region of 4000 cm^−1^–600 cm^−1^, collecting 16 scans with 4 cm^−1^ spectral resolution.

A Contact Angle Meter (Kyowa Surface Chemistry Co., Ltd., Tokyo, Japan) and KSV Instruments CAM 100 equipment (KSV Instruments, Helsinki, Finland) were used to measure static contact angles. A 10 μL droplet of distilled water was applied to the membrane’s surface to measure the water contact angle at room temperature. To reduce experimental mistakes, an average of three measurements ± standard deviation was provided. The surface morphologies of the polymeric membranes were observed by scanning electron microscopy (SEM) on an FEI electron microscope (Thermo Fisher, Eindhoven, The Netherlands). Secondary electron imaging was conducted at an accelerating voltage of 30 keV coupled with energy-dispersive X-ray spectroscopy (EDS) for elemental analysis identification. The small pieces of polymeric membranes were cut, sputter-coated with a thin gold layer, mounted on a stub, and placed in the microscope’s chamber.

#### 3.3.2. Water Retention and Porosity of the Polymeric Membranes

The water retention capacity and porosity of the polymeric membranes were determined by the gravimetric method to determine whether they can be used in a membrane separation system for treating pollutants in waste-water: in this case, Cd^2+^. The polymeric membranes were cut into small pieces, wiped with filter paper to remove any excess of distilled water, and then dried in an oven at 60 °C for 6 h. After and before, the obtained polymeric membranes were weighed using an analytical balance KERN ADB 100-4 (Kern & Sohn GmbH, Balingen, Germany).

The degree of water retention capacity (WR (%)) was determined by using Equation (1) [31]:

(1) $$WR\left(\%\right)=\frac{m_{wet}-m_{dry}}{m_{wet}}\times 100$$

The porosity (ε (%)) of polymeric membranes was determined using Equation (2) [31].

(2) $$\varepsilon \left(\%\right)=\frac{m_{wet}-m_{dry}}{A\cdot \rho_{dw}\cdot \sigma }\times 100$$

where m_wet_ and m_dry_—the weight of wet and dry polymeric membranes (g); A—the area of the polymeric membrane (cm^2^), ρ_dw_—the distilled water density, and σ—the thickness of the polymeric membrane (cm).

#### 3.3.3. Thermal Stability Investigation of the Polymeric Membranes

The TGA measurements of the polymeric membranes were studied using the TGA Q5000 IR system (TA Instruments, New Castle, DE, USA). The measurements for the samples with a mass of approximately 5 mg were conducted under nitrogen flow (99.99%, 50 mL/min) at 10 °C/min in platinum pans (100 μL) from 30 °C to 700 °C.

#### 3.3.4. Impedance Spectroscopy Investigation of the Polymeric Membranes

The ionic conductivity of the polymeric membranes was determined using a four-electrode alternating current (a.c.) impedance method with a frequency range of 0.01 Hz to 100 MHz, an a.c. voltage of 10 mV perturbation and a 0.0 V DC rest voltage. Impedance spectra were obtained using a Princeton Applied Research Model 2273 Advanced Electrochemical System (Princeton, NJ, USA). The polymeric membranes were secured in a custom-designed measurement cell comprising four electrodes: two outer silver wires served as the working and counter electrodes, while two inner silver wires functioned as reference electrodes. Conductivity measurements were performed under fully hydrated conditions with the cell filled with distilled water at room temperature.

To calculate the ionic conductivity of the polymeric membranes, we use the fact that the total resistance (R_t_) includes two components: the bulk membrane resistance (R_b_) and the charge transfer resistance (R_ct_). The total resistance can be expressed as (1) [22,32]:

(3) $$R_{t}=R_{b}+R_{ct}$$

Then, the charge transfer resistance is (4) [32]

(4) $$R_{ct}=R_{t}-R_{b}$$

The charge transfer resistance (R_ct_) is a critical parameter in electrochemical systems particularly in processes involving electrochemical reactions. It quantifies the resistance to charge transfer at the interface between an electrode and an electrolyte during a redox reaction. Electrochemical impedance spectroscopy (EIS) is the most widely used and effective technique for determining R_ct_. This method applies an a.c. voltage to the electrochemical cell and measures the impedance over a range of frequencies. The Nyquist plot generated from the data often exhibits a semicircular arc, where the diameter corresponds to R_ct_.

The ionic conductivity (σ) of the membrane was calculated using the following Equation (5) [22,32]:

(5) $$\sigma =\frac{L}{R_{b}\cdot A}$$

where L—the thickness of the polymeric membrane (0.0009 m); R_b_—the resistance of the polymeric membrane; and A—the cross-sectional area of the polymeric membrane (6.35 × 10^−5^ m^2^) (L/A = 14.17 m^−1^).

## 4. Results and Discussion

### 4.1. Electrochemical Separation System Performance

In order to establish the optimal operating parameters of the electrochemical separation system, a series of preliminary tests was performed. As a result of these tests, it was found that a low applied voltage at the lead electrodes of the electrochemical separation system (<10 V) leads to a low removal rate of cadmium ions, and a voltage over 10 V leads to the cracking of polymeric membranes and increases energy consumption. Additionally, a concentrated synthetic waste-water (over 1500 ppm) indicates that high energy consumption was higher and also led to damage and clogging of the polymeric membranes. All of these aspects are not beneficial and advantageous, because they lead to increased material consumption, economic costs, and energy consumption. Additionally, after testing the electrochemical separation system for more than 1.5 h of operation, it was observed that the polymer membranes were unable to withstand the process, resulting in a very high specific energy consumption. In this work, only the best results obtained using the electrochemical separation system with the prepared polymeric membranes were included.

All electrochemical separation tests for both types of polymeric membranes (CA-PEG-PVST0 and CA-PEG-PVS0 membranes) were completed under the same optimal operating conditions: a constant applied voltage of 10 V, operation time of 1.5 h, at room temperature, and without the recirculation of synthetic waste-water. After the electrochemical separation test, the polymeric membranes were noted as CA-PEG-PVST1 (polymeric membrane with TiO_2_ nanoparticles) and CA-PEG-PVS1 (polymeric membrane without TiO_2_ nanoparticles).

The electrochemical separation system performance and the efficiency of the polymeric membranes were evaluated by the removal rate of the cadmium ions (R_Cd_^2+^, %), the current efficiency (CE, %), the mass flow of cadmium ions (J_Cd_^2+^ gm ^−2^ h^−1^), the specific energy consumption (SEC, kWh L^−1^), and the mass transfer coefficient (k).

The R_Cd_^2+^, CE, SEC, J_Cd_^2+^, and k were calculated using the following Equations (6)–(10) [22,23,25,26]:

(6) $$R_{{Cd}^{2+}}(\%)=\frac{C_{i}-C_{f}}{C_{i}}\times 100$$

(7) $$CE\;\left(\%\right)=\frac{\left(C_{i}-C_{f}\right)\cdot z\cdot F\cdot V}{\bar{I}\cdot t\cdot M}\times 100$$

(8) $$J_{{Cd}^{2+}}(g\;m^{-2}h^{-1})=\frac{\left(C_{i}-C_{f}\right)\cdot V}{A\cdot t}$$

(9) $$SEC\;(kWh\;L^{-1})=\frac{U\cdot \int_{0}^{t}Idt}{V}$$

(10) $$ln\left(\frac{C_{f}}{C_{i}}\right)=-k\cdot \left(\frac{A}{V}\right)\cdot t$$

where C_i_—the initial concentration of Cd^2+^ before the electrodialysis test (g L^−1^); C_f_—the final concentration of Cd^2+^ from the dilute compartment after 1.5 h of electrodialysis test (g L^−1^), U—the applied voltage between the anode and cathode (V), F—Faraday’s constant (96,486 A s mol^−1^); $\bar{I}$—the average electrical current (A); t—the experimental time (s), z—the ionic valence state of Cd^2+^, M—the molar mass of Cd^2+^ (g mol^−1^); A—the effective working area of the polymeric membrane (m^2^); V—total volume of solution from all compartment (L); k—the mass transfer coefficient.

Considering the removal rate of the cadmium ions, current efficiency, mass flow of cadmium ions, specific energy consumption, and mass transfer coefficient, 10 V was the optimal applied voltage: the removal rate values of Cd^2+^ were 95.53% for the CA-PEG-PVST1 membrane and 85.29% for the CA-PEG-PVS1 membrane after 1.5 h (Table 1). The introduction of TiO_2_ nanoparticles into the polymeric membrane matrix improves the removal rate of the cadmium ions, which may be due to the changes in the structure of the selective membrane layer (less dense, small pore size, large numbers of aggregates, and agglomeration of nanoparticles) [7,17]. The high removal rate of the CA-PEG-PVST1 membrane can be attributed to the strong chains formed between polymers, copolymers, and TiO_2_ nanoparticles within the polymeric membrane matrix via hydrogen bonds. Also, the CA-PEG-PVST1 membrane exhibits high removal capacity for cadmium compared to the CA-PEG-PVS1 membrane, which is possibly due to its large surface area, more active sites and electrostatic force. The lower value of the removal rate obtained for the CA-PEG-PVS1 membrane can be attributed to its larger pore size and, possibly, to the reduction of the hydration layer in the polymeric membrane. These results are confirmed by SEM and FTIR analysis. From Table 1, it can be seen that the values for the current efficiency and the mass flow of cadmium ions are high, while the specific energy consumption decreases for the CA-PEG-PVST1 membrane. These can be attributed to the increase in the migration rate and the relative mobility of cadmium ions in the solutions from the compartments (anodic-central and central-cathodic). The small difference in values of the mass flow of cadmium ions (1.29 g m^−2^ h^−1^ for the CA-PEG-PVST1 membrane and 1.14 g m^−2^ h^−1^ for the CA-PEG-PVS1 membrane) can be due to the low amount of TiO_2_ nanoparticles incorporated into the polymeric membrane matrix. The results indicate that incorporating TiO_2_ nanoparticles into the polymeric membrane matrix significantly improves the rejection of cadmium ions from synthetic waste-water using the electrochemical separation system. Min et al. [7] used an electrodialysis device including an ion-exchange membrane for the treatment and recovery of Cd^2+^ from a zinc smelting waste-water that contains high concentrations of Cd^2+^. They related that the higher cadmium treatment efficiency was 85.4% and the recovery rate was 52.6% at an applied voltage of 50 V. Lee [24] investigated the removal of cadmium from synthetic waste-water containing cadmium in high concentrations by an electrodialysis system and cation- and anion-exchange membranes at different operating conditions. They reported that the removal rate of cadmium depends on the flow rate and the applied voltage on the electrodialysis system. The higher removal rate of cadmium ions (99.99%) from the diluted solution was obtained at a flow rate of 3.2 L min^−1^ after 120 min. Nile et al. [33] related that the mass balance of cadmium in the Muharram Aisha waste-water treatment plant was 4832.44 g day^−1^ in treated waste-water and 8164.52 g day^−1^ in sludge. This indicated that the mixed suspended solids were the most sensitive factor. Cadmium sensitivity was analyzed by mixed suspended solids in the extended aeration system. The results indicated that the higher the mixed suspended solids concentration (mg L^−1^), the higher the cadmium removal from treated waste-water. It was found that increasing mixed suspended solids by a biological treatment method reduced the cadmium concentration. For 5 months, the treatment plant was subsequently operated with the mixed suspended solids increased from 1500 to 4500 mg L^−1^, which reduced the cadmium concentration in the waste-water from 0.36 to 0.01 mg L^−1^.

**Figure 3:**
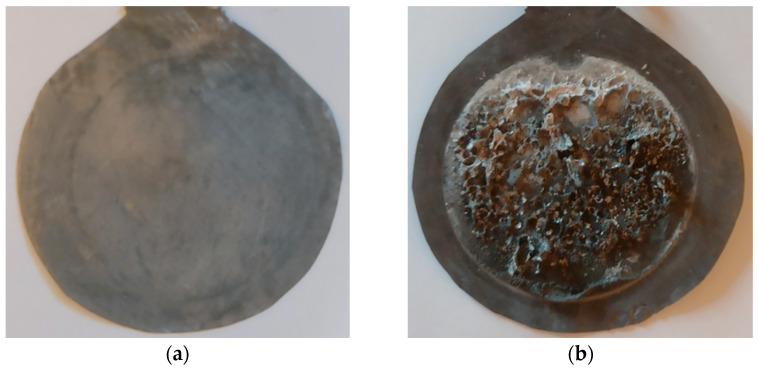
The macroscopic image of the cathode lead electrodes: (**a**) pure lead electrode before the electrochemical separation test; (**b**) metallic cadmium deposits on the lead electrode after the electrochemical separation test.

The metallic cadmium deposits on the lead electrode (cathode) can be observed when the electrochemical separation system is disassembled after the experiment is completed. It is possible that a small amount of TiO_2_ nanoparticles will be removed from the polymeric membrane and will also end up on the cathode electrode (Figure 3).

The macroscopic image shows that the cadmium metal deposits on the lead electrode are not compact. These deposits can be used in various domains, such as electro-galvanization; the manufacture of batteries, semiconductors, alloys, control rods in nuclear reactors, infrared detectors, stained glass, mirrors, pigments and dyes; in the production of solar cells; and as a pigment in certain paints and plastics.

### 4.2. FTIR, SEM and EDS Analysis of the Polymeric Membranes

**Figure 4:**
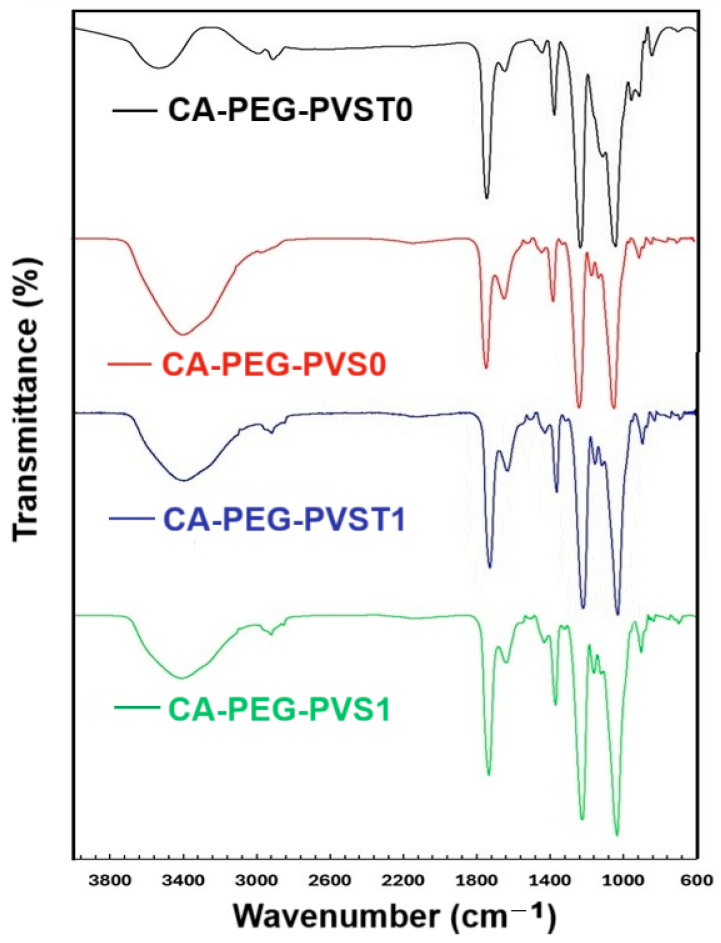
FTIR spectra of polymeric membranes with and without TiO_2_ nanoparticles before and after the electrochemical separation test.

The obtained polymeric membranes (CA-PEG-PVST0 and CA-PEG-PVS0 membranes) and the uptake of cadmium ions (CA-PEG-PVST1 and CA-PEG-PVS1 membranes) by them were investigated by FTIR analysis (Figure 4).

In untested polymeric membranes (before the electrochemical separation test), the characteristic peaks of the stretching vibration of the O-H group appeared at 3528 cm^−1^ (CA-PEG-PVST0 membrane) and at 3391 cm^−1^ (CA-PEG-PVS0 membrane), and in tested polymeric membranes (after the electrochemical separation test), this appeared at around 3400 cm^−1^ (CA-PEG-PVST1 and CA-PEG-PVS1 membranes). The significant shift in the polymeric membrane (CA-PEG-PVST0 membrane) from 3528 cm^−1^ to 3391 cm^−1^ suggests that the presence of TiO_2_ nanoparticles led to the association of water molecules. Comparing the IR spectra of polymeric membranes before and after the electrochemical separation test (CA-PEG-PVST0 and CA-PEG-PVST1 membranes), the O-H band shifting from 3528 cm^−1^ to 3400 cm^−1^ suggests that cadmium ions could coordinate with the hydroxyl groups around TiO_2_ nanoparticles. In the case of the polymeric membranes, the O-H band shift was also recorded before and after the electrochemical separation test (CA-PEG-PVS0 and CA-PEG-PVS1 membranes). Still, it was much smaller than in the case of polymeric membranes with TiO_2_ nanoparticles, suggesting that cadmium ions bound the hydroxyl groups of PEG and CA, confirming the formation of a coordination complex.

In all polymeric membranes, the aromatic stretching vibrations of -CH groups appeared at around 3000 cm^−1^. The absorption band attributed to the stretching vibration of C=O from CA was observed in all membranes as intense peaks at approximately 1735 cm^−1^, indicating an O–H⋯C=O interaction between CA and PEG [34,35].

Analyzing the band corresponding to C=N in pyridine rings from the poly(4-vinylpyridine) blocks, specific peaks were registered in all polymeric membranes, before and after the electrochemical separation test, as follows: 1645 cm^−1^ in the CA-PEG-PVST0 membrane, 1636 cm^−1^ in the CA-PEG-PVS0 membrane, 1636 cm^−1^ in the CA-PEG-PVST1 membrane, and 1640 cm^−1^ in the CA-PEG-PVS1 membrane. Additionally, a significant shift from 1645 cm^−1^ to 1636 cm^−1^ was observed in the case of the CA-PEG-PVST1 membrane after cadmium ions sorption, indicating the formation of a coordination complex in which cadmium ions bind to the nitrogen groups of poly(4-vinylpyridine).

The stretching vibration of C=N in the pyridine ring registered at 1417 cm^−1^ from the poly(4-vinylpyridine-co-styrene) before the electrochemical separation test was shifted to 1431 cm^−1^ in the polymeric membrane without TiO_2_ nanoparticles after the electrochemical separation test (CA-PEG-PVS0 and CA-PEG-PVS1 membranes) and to 1436 cm^−1^ in the polymeric membranes with TiO_2_ nanoparticles after the electrochemical separation test (CA-PEG-PVST0 and CA-PEG-PVST1 membranes). The more significant shifting in the case of the CA-PEG-PVST0 and CA-PEG-PVST1 membranes suggests the coordination of Ti ions with N atoms of the pyridine ring from poly(4-vinylpyridine).

The absorption band at 1160 cm^−1^ is associated with the C–O–C vibrations of CA. In the case of the polymeric membrane (CA-PEG-PVST0 membrane), a shoulder was observed at 1160 cm^−1^ before the electrochemical separation test. However, after the electrochemical separation test, the spectrum of the polymeric membrane (CA-PEG-PVST0 membrane) presented an intense peak at 1160 cm^−1^. This may indicate that the interaction with cadmium ions is responsible for modifying this band. In the case of the polymeric membranes, before and after the electrochemical separation test (CA-PEG-PVS0 and CA-PEG-PVS1 membranes), a peak is observed at the same wavenumber (1160 cm^−1^) that is slightly more intense in the case of the polymeric membrane (CA-PEG-PVS1 membrane), which is probably due to the interaction of cadmium ions.

The absorption band at approximately 960 cm^−1^ may reflect the C–O–C vibrations of the ether groups of CA as well as the C–O vibrations of the ether bonds of PEG present in the composition of the obtained polymer membranes. In the case of the polymeric membranes without TiO_2_ nanoparticles, before and after the electrochemical separation test (CA-PEG-PVS0 and CA-PEG-PVS1 membranes), a peak was recorded at the same wavenumber (954 cm^−1^). In the case of the polymeric membrane, after the electrodialysis test (CA-PEG-PVS1 membrane), as a result of interactions with cadmium ions, this band became less intense than in the case of the polymeric membrane before the electrochemical separation test (CA-PEG-PVS0 membrane). When TiO_2_ nanoparticles were added into the composition of the polymeric membranes, a peak was recorded at 949 cm^−1^ in the case of the polymeric membrane before the electrochemical separation test (CA-PEG-PVST0 membrane). After the electrochemical separation test, the IR spectrum of the polymeric membrane (CA-PEG-PVST1 membrane) recorded a slightly less intense peak at 954 cm^−1^. The band shift, as well as the decrease in its intensity, showed that the interaction with the cadmium ions modifies the vibrations of the O-Ti-O bonds [34,35,36].

Depending on the presence or absence of TiO_2_ nanoparticles in the membranes’ composition, as well as whether or not the membrane passed through an electrochemical separation system, the water contact angle values showed a notable variation in the wetting properties of the membranes.

Starting from the CA-PEG-PVS0 membrane, which presents a contact angle value of 55.53°, the inclusion of TiO_2_ nanoparticles decreases it to 21.5° for the CA-PEG-PVST0 membrane, indicating a more pronounced hydrophilic character. This behavior can be explained by the coordination of Ti ions with nitrogen atoms of the pyridine ring in the poly(4-vinylpyridine) blocks. After these membranes were subjected to the electrochemical separation system, the contact angle increased to 65.72° for the CA-PEG-PVS1 membrane and to 63.5° for the CA-PEG-PVST1 membrane. This increase in the contact angle values, and implicitly the hydrophobicity of the membranes, after the electrochemical separation system, can be explained by the retention of cadmium ions from synthetic waste-water. In both cases, the N atoms from the pyridine ring of the poly(4-vinylpyridine) blocks established coordination connections with cadmium ions. This Cd^2+^←:N coordination decreased the hydrophilicity of the membranes by saturating the polar groups. Additionally, TiO_2_ nanoparticles-containing membranes enable further cadmium ions adsorption. The binding of cadmium ions to pyridine groups or their anchoring by adsorption to TiO_2_ nanoparticles resulted in a surface with lower availability to form hydrogen bonds with water.

Table 2 shows the water retention and porosity values of the prepared polymeric membranes with and without TiO_2_ nanoparticles (CA-PEG-PVST0 and CA-PEG-PVS0 membranes).

The higher water retention and porosity values obtained for the polymeric membrane (CA-PEG-PVST0 membrane) confirm the influence and the role of TiO_2_ nanoparticles in the polymeric membrane matrix. The obtained values confirmed that the polymeric membrane with TiO_2_ nanoparticles (CA-PEG-PVS0 membrane) is more hydrophilic and porous compared to the polymeric membrane without TiO_2_ nanoparticles (CA-PEG-PVS0 membranes).

**Figure 5:**
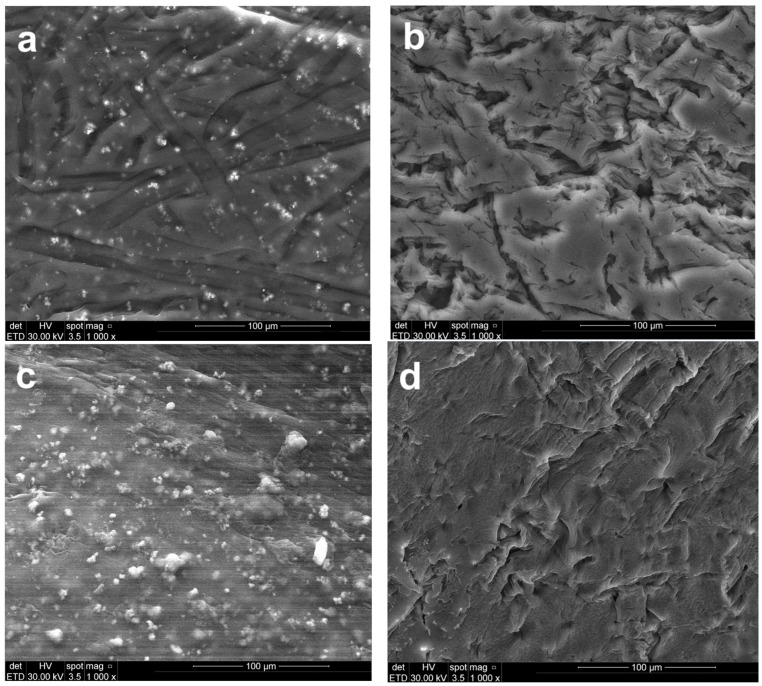
SEM images of the top view of the polymeric membranes: (**a**) CA-PEG-PVST0 membrane (before the electrochemical separation test), (**b**) CA-PEG-PVS0 membrane (before the electrochemical separation test), (**c**) CA-PEG-PVST1 membrane (after the electrochemical separation test), and (**d**) CA-PEG-PVS1 membrane (after the electrochemical separation test).

**Figure 6:**
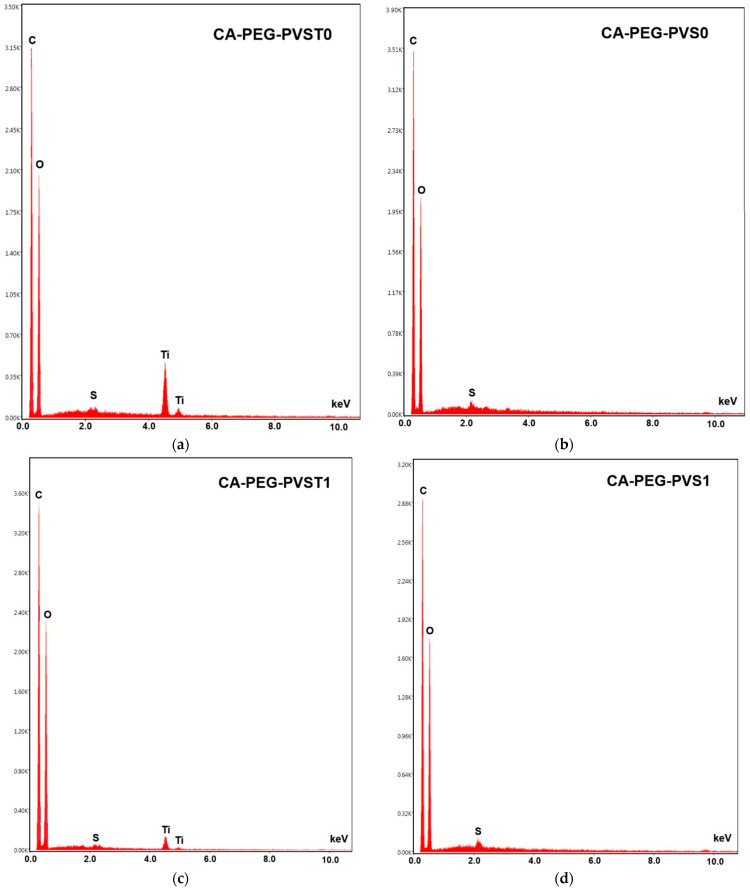
EDS analysis of the polymeric membranes: (**a**) CA-PEG-PVST0 membrane (before the electrochemical separation test), (**b**) CA-PEG-PVS0 membrane (before the electrochemical separation test), (**c**) CA-PEG-PVST1 membrane (after the electrochemical separation test), and (**d**) CA-PEG-PVS1 membrane (after the electrochemical separation test).

The surface morphology of the polymeric membranes, both prior to and following the electrodialysis test, was characterized using SEM (Figure 5). Changes in the surface elemental composition associated with electrochemical separation were evaluated through EDS analysis (Figure 6).

The CA-PEG-PVST0 membrane (Figure 5a) exhibits a compact and relatively uniform surface distinguished from the TiO_2_ nanoparticles-free system by its finer texture and minimal topographical irregularities. Bright nanoscale particulates visible in the SEM correspond to TiO_2_ nanoparticles, which is confirmed by the distinct Ti peaks (~4.5–4.8 keV) in the EDS spectrum (Figure 6a). The dominant C and O peaks originate from the CA/PEG matrix, while the weaker S peaks likely reflect the residual processing components. The uniform surface morphology is consistent with the synthesis conditions: the incorporation of TiO_2_ nanoparticles increases the viscosity of the casting solution, slowing solvent–nonsolvent exchange during phase inversion. This delayed demixing mechanism promotes the formation of a denser skin layer, resulting in a smoother and more compact top surface after solidification.

The micrographs of CA-PEG-PVST1 and CA-PEG-PVS1 membranes (Figure 5b,d) showed rough surface morphologies with macro-voids and large pores compared to CA-PEG-PVST0 and CA-PEG-PVS1 membranes (Figure 5a,c). The relatively smoother micro-scale surface morphology of samples containing TiO_2_ nanoparticles has a smaller pore size and lower agglomeration compared to that of samples containing TiO_2_ nanoparticles. In the absence of TiO_2_ nanoparticles, the CA-PEG-PVS0 membrane (Figure 5b) shows a rougher and more heterogeneous surface with pronounced ridges and valley-like features. These morphological characteristics are typical of a faster demixing process, resulting from the lower viscosity of the TiO_2_ nanoparticles-free casting solution. Rapid solvent–nonsolvent exchange during phase inversion leads to a less compact skin layer and more pronounced surface roughness. EDS spectra display only C, O, and a small S contribution, with no Ti peaks, confirming the absence of inorganic filler (Figure 6b). The contrast between CA-PEG-PVS0 and CA-PEG-PVST0 highlights the structural role of TiO_2_ nanoparticles in producing a more stabilized, compact skin layer and a smoother final surface.

After the electrochemical separation test, the CA-PEG-PVST1 membrane (Figure 5c) undergoes the most significant surface modification among all samples. SEM reveals extensive fine particulate deposition and a higher density of bright contrast features across the surface. EDS continues to show clear Ti peaks, demonstrating that the TiO_2_ nanoparticles remain exposed on the surface even after the electrochemical separation test (Figure 6c). A slightly elevated spectral background suggests the presence of additional surface deposits or fouling layers accumulated during operation. Compared with the CA-PEG-PVST0 membrane, the CA-PEG-PVST1 membrane surface displays reduced visible pore openings and partial masking of the underlying polymer texture. These changes indicate pore narrowing, partial pore blockage, and surface coverage by deposit layers. This behavior aligns with the high Cd^2+^ removal efficiency of the CA-PEG-PT1 membrane and suggests that the TiO_2_ nanoparticles domains serve as active interfacial sites for ionic interactions and deposit formation during electrochemical separation.

Although Cd peaks are not detected in the EDS spectra, the morphological evolution strongly supports TiO_2_ nanoparticles-mediated adsorption or clustering phenomena on the surface.

The CA-PEG-PVS1 membrane (Figure 5d) retains much of the surface roughness characteristic of CA-PEG-PVS0 but exhibits moderate deposition after the electrochemical separation test. SEM shows scattered small particles and localized surface fouling, but no uniform layer or extensive particulate networks as observed in CA-PEG-PVST1. EDS reveals only C, O, and S peaks, confirming that no TiO_2_ nanoparticles are present (Figure 6d). The lower degree of surface modification relative to CA-PEG-PVST1 corresponds with the membrane’s lower Cd^2+^ removal efficiency and indicates that fouling results primarily from general electrochemical separation operation, not nanoparticle-mediated interactions. Without TiO_2_ nanoparticles, the membrane lacks the inorganic adsorption sites that contribute to more extensive deposit formation, resulting in a surface that remains largely like its pre-electrochemical separation state (similar to the principle of electrodialysis process).

Figure 6 shows the distribution of the chemical elements for C, O, S, and Ti. From the figure, the peaks of C (around 0.2 keV), O (around 0.25 keV), and S elements (around 2.1 keV) can be observed, indicating the characteristic constituents present in all polymeric membranes (before and after the electrochemical separation test). The new peaks of the Ti element (4.5 keV and 5 keV) were clearly observed for the CA-PEG-PVST0 and CA-PEG-PVST1 membranes (before and after the electrochemical separation test), indicating the characteristic constituent of TiO_2_. These characteristic peaks indicated the successful incorporation of TiO_2_ nanoparticles into the polymeric membrane matrix [36]. It can also be observed that the intensity of Ti peaks decreases in the polymeric membrane tested in the electrochemical separation system (CA-PEG-PVST1 membrane), which is possibly due to the physical adsorption of cadmium ions to the TiO_2_ nanoparticles in the matrix layer of the polymeric membrane [36,37,38].

### 4.3. Thermal Analysis of the Polymeric Membranes

**Figure 7:**
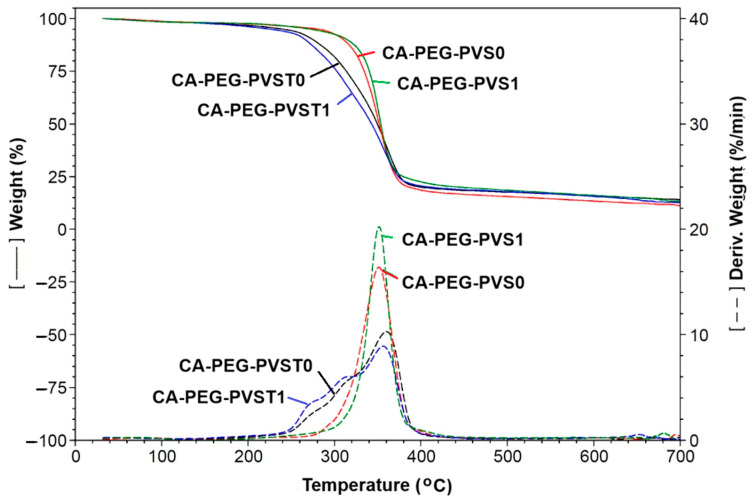
TGA and the corresponding temperature derivative curves of polymer membranes before and after the electrochemical separation tests: CA-PEG-PVST0 membrane (before the electrochemical separation test), CA-PEG-PVS0 membrane (before the electrochemical separation test), CA-PEG-PVST1 membrane (after the electrochemical separation test), and CA-PEG-PVS1 membrane (after the electrochemical separation test).

The TGA curves, the temperature derivative curves, and the maximum decomposition temperature (T_max_) values of the prepared polymeric membranes (before and after the electrochemical separation tests) are indicated in Figure 7 and Table 3.

The TGA curve of the polymeric membranes showed three major weight loss stages (Figure 7). The first weight loss that occurred at 35–135 °C can be due to the removal of bound water or loss of adsorbed water in the polymeric membrane matrix. At the second stage, the weight loss region of 135–500 °C can be attributed to the deacetylation reaction correlated with the elimination reactions of water and also to the decomposition of CA and PEG [34,35,36,37]. The third stage was between 500 °C and 700 °C, and it was associated with the carbonization and decomposition of the polymeric membranes to the ash. The enhanced thermal stability of the polymeric membranes can be due to the interaction between the chains of the mixture of the polymers (CA and PEG), the copolymer, and TiO_2_ nanoparticles (Table 3). The incorporation of TiO_2_ nanoparticles improved the thermal stability of the polymeric membrane [38,39,40]. Before the electrochemical separation test, the maximum decomposition temperature (T_max_) values were 360 °C for the CA-PEG-PVST0 membrane and 351.5 °C for the CA-PEG-PVS0 membrane. After the electrochemical separation test, the T_max_ values decreased, possibly due to the decrease in the CA/PEG and copolymer chains’ mobility, which can occur in the polymeric membranes (CA-PEG-PVST1 and CA-PEG-PVS1 membranes). However, the CA-PEG-PVST1 membrane presented a higher maximum decomposition temperature compared to the CA-PEG-PVS1 membrane. This showed that the coordination of Cd^2+^ with the polymers, copolymer, and TiO_2_ nanoparticles improved the thermal stability. In the case of the CA-PEG-PVST1 membrane, the T_max_ value decreased after the electrochemical separation test, which is possibly due to the hydrogen bonds of the hydroxyl groups on TiO_2_ nanoparticles with waste-water that contains cadmium ions. The difference in T_max_ values for the polymeric membranes that contain TiO_2_ nanoparticles, before and after the electrochemical separation test (CA-PEG-PVST0 and CA-PEG-PVST1 membranes), can be due to the strong binding of hydroxyl groups to the TiO_2_ nanoparticles and the mobility of Cd^2+^. The difference in the residue at 700 °C can be attributed to the presence of TiO_2_ nanoparticles in the polymeric membranes. The residue mass value decreased for the CA-PEG-PVST1 membrane after the electrochemical separation test, which is possibly due to the lower moisture and volatile matter content. Additionally, it may be attributed to the cadmium particles retained in the polymer matrix of the polymeric membrane or adsorbed on the surface of the TiO_2_ nanoparticles. These results are corroborated by the FTIR analysis and the obtained values for the removal rate and mass flow of Cd^2+^. The obtained polymeric membranes present a high thermal stability. Aparicio et al. [38] fabricated a composite polymer membrane based on polyvinyl alcohol/TiO_2_ nanoparticles (composition of 1:12%) cross-linked with glutaraldehyde. They reported that the decomposition temperature of this membrane occurs around 250 °C. Shafiq et al. [39] reported that the thermal stability increased for the composite membranes containing cellulose acetate/polyethylene glycol and a high concentration of TiO_2_ nanoparticles (25 wt.%).

### 4.4. Impedance Spectroscopy Analysis of the Polymeric Membranes

Understanding and controlling R_ct_ is essential for optimizing the performance of electrochemical devices, minimizing energy losses, and enhancing efficiency.

When comparing membranes with different ionic conductivities (σ), the behavior of each membrane in terms of ion transport, energy losses, and overall performance in applications (like fuel cells or separation processes) can be interpreted based on the magnitude of their electrical conductivities.

**Figure 8:**
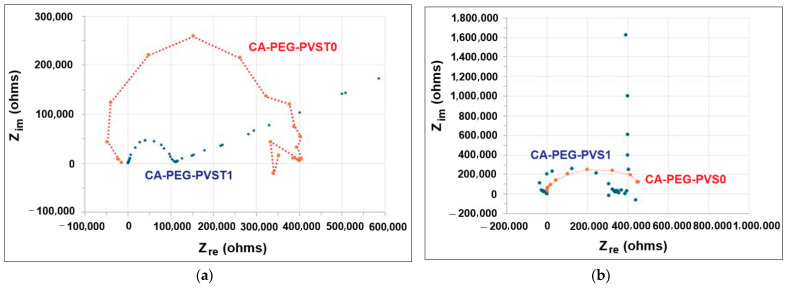
The impedance spectra of the polymeric membranes: (**a**) with TiO_2_ nanoparticles; CA-PEG-PVST0 and CA-PEG-PVST1 membranes (before and after the electrochemical separation test); (**b**) without TiO_2_ nanoparticles; CA-PEG-PVS0 and CA-PEG-PVS1 membranes (before and after the electrochemical separation test).

Figure 8 presents the spectra of the polymeric membranes before and after the electrochemical separation tests.

The electrochemical impedance spectra recorded for the polymeric membranes, before and after the electrochemical separation tests, showed fully resolved semicircles. The absence of a linear part for the initial polymeric membranes, with and without TiO_2_ (CA-PEG-PVST0 and CA-PEG-PVS0 membranes), showed that the exchange process occurred without diffusional control. Furthermore, the presence of a single semicircle indicates a unique relaxation process. However, after the electrochemical separation test, the a.c. impedance spectrum of the polymeric membrane without TiO_2_ nanoparticles (CA-PEG-PVS1 membrane) put into evidence a capacitive behavior at low frequencies (Figure 8b), while the polymeric membrane with the TiO_2_ nanoparticles incorporated (CA-PEG-PVST1 membrane) revealed a diffusional control process (Figure 8a).

The electrical parameters characteristic of the studied polymeric membranes are presented in Table 4. Variations in ion mobility within the polymeric membranes result in modifications to the membrane resistance.

The results presented in Table 4 indicate that the addition of TiO_2_ nanoparticles to the CA-PEG polymeric matrix generates an increase in the ionic conductivity. A significant increase in the ionic conductivity values for the polymeric membrane with included TiO_2_ nanoparticles was recorded after the electrochemical separation test (CA-PEG-PVST1 membrane) (21.963 mS m^−1^). It could be attributed to the aggregation of the cadmium ions on the surface of polymeric membrane, which may lead to the formation of ion clusters that may reduce the number of mobile charge carriers. In the meantime, the CA-PEG-PVS0 membrane with ionic conductivity of 1.771 mS m^−1^ offers moderate performance compared to the CA-PEG-PVST0 membrane (2.610 mS m^−1^). Such a polymeric membrane (CA-PEG-PVS0 membrane) can be suitable for less demanding applications where ion transport is still important but not as critical as in high-performance devices. The CA-PEG-PVS1 membrane has an ionic conductivity of 1.388 mS m^−1^, which would be the least efficient in terms of ion transport and energy loss. However, it might be useful in applications where ionic conductivity is less important but other properties (e.g., selectivity, mechanical strength, or chemical resistance) are prioritized. Aparicio et al. [38] reported that the obtained membranes based on polyvinyl alcohol and nano-sized TiO_2_ fillers (1:12%) have an ionic conductivity value of 0.016 S cm^−1^ at 130 °C. Sugumaran et al. [41] prepared a membrane based on polyvinylidene fluoride-co-hexafluoropropylene, cellulose acetate, and TiO_2_ nanoparticles. They reported that the membrane presents an ionic conductivity of 0.0319 mS m^−1^. Lee et al. [42] fabricated composite membranes containing poly(arylene ether ketone) and different amounts of functionalized TiO_2_ nanoparticles (1, 3, 5, 7, and 9 wt%). The highest ionic conductivity (0.746 mS m^−1^) was obtained for the membrane with 5 wt% functionalized TiO_2_ nanoparticles. Bae et al. [43] reported that the synthesized poly(arylene ether sulfone ketone) multiblock copolymer membranes have an ionic conductivity of 30 mS cm^−1^ at 80 °C.

The initial bulk resistance of the polymeric membrane with TiO_2_ nanoparticles (CA-PEG-PVST1) is noticeably lower compared to the polymeric membrane without TiO_2_ nanoparticles (CA-PEG-PVS1) subjected to Cd^2+^ retention. The observed increase in polymeric membrane resistance (CA-PEG-PVS1 membrane) can be attributed to the retention of Cd^2+^ within the polymeric membrane structure. However, in the case of the CA-PEG-PVST1 membrane, the resistance (bulk membrane resistance (R_b_) and charge transfer resistance (R_ct_)) are much lower after the electrochemical separation test. When the membrane retains metal ions, like Cd^2+^, these ions often remain in or on the membrane matrix depending on the membrane structure, thus increasing the number of mobile charge carriers. So, being involved in more ions than in a higher ionic conductivity material results in lower electrical resistance.

Cadmium ions are divalent cations and can significantly contribute to ionic conductivity. Once they are retained in the membrane with ion exchange or adsorption capabilities, they increase the overall concentration of mobile ions within or near the membrane structure, thereby enhancing ion transport and leading to lower resistance. As the membrane has functional groups, carboxyl groups, the Cd^2+^ ions are able to displace other ions. Then, they could bind or be partially mobile within the membrane. As a result, the membranes provide a more conductive ionic environment. Often, Cd^2+^ replaces ions with lower mobility or charge, making the membrane more conductive. Cadmium ions can interact strongly with the membrane matrix. This interaction may cause the polymer chains to swell or slightly open up, even altering pore size or connectivity. Improving pathways for ion transport enables the reduction in electrical resistance. In the meantime, it is likely that in the porous membranes, Cd^2+^ ions may form weakly bound surface complexes, while a layer of Cd^2+^- associated species (e.g., Cd(OH)^+^) may act as a more conductive interfacial layer than the bare membrane surface. However, it should be mentioned that over time or at higher concentrations, the cadmium ions can also foul or saturate the membrane. That could eventually increase resistance again. Materials with higher ionic conductivity or greater catalytic activity typically exhibit lower R_ct_ values, as they facilitate faster charge transfer. Additionally, a higher surface area provides more active sites for charge transfer, while surface modifications or catalysts can further reduce R_ct_ by enhancing reaction kinetics at the electrode surface. Conversely, systems involving complex, multi-step reactions, such as those in fuel cells or certain batteries, may display higher R_ct_ values due to the slower charge transfer processes. The value of R_ct_ is also influenced by the concentration of reactive species (oxidants or reductants). Lower concentrations can lead to an increase in R_ct_, as fewer molecules are available to participate in the charge transfer process.

**Figure 9:**
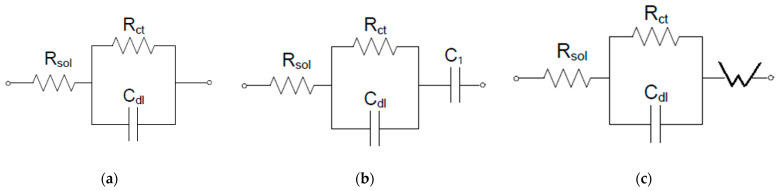
Equivalent electrical circuit for a simplified interface: (**a**) CA-PEG-PVS0 and CA-PEG-PVST0 membranes; (**b**). CA-PEG-PVS1 membrane; (**c**) CA-PEG-PVST1 membrane. R_sol_—the resistance of the solution; R_ct_—the charge transfer resistance, C_dl_—the double layer capacitance; C_1_—in series capacitance; W—Warburg impedance.

The Nyquist diagrams were modelled considering a Randles-type circuit having a simplified interface and a single time constant, according to Figure 8, for the initial polymeric membranes (CA-PEG-PVST0 and CA-PEG-PVS0 membranes). To model the behavior of the polymeric membranes (CA-PEG-PVST1 and CA-PEG-PVS1 membranes), which were exposed to a solution containing cadmium ions, a supplementary element, a capacitive one, was introduced in series. For the CA-PEG-PVST1 membrane, a Warburg impedance allowed the electrical modelling of the equivalent circuit (Figure 9).

The Nyquist plot for a polymeric membrane with an ionic conductivity of 21.963 mS m^−1^ (CA-PEG-PVST1 membrane), where a semicircle is followed by a straight line with a slope of 30 degrees extending up to 583.870 kohm, suggests the presence of both charge transfer resistance and diffusion-related processes. The present semicircle (112.903 kohm) typically represents the charge transfer resistance R_ct_ and the double-layer capacitance. The diameter of the semicircle indicates the magnitude of R_ct_. The fact that the semicircle ends at 112.903 kohm means that the charge transfer resistance is approximately 112.258 kohm. This value reflects the resistance associated with the charge transfer process at the membrane/electrode interface, which in this case seems relatively high, and is consistent with the medium ionic conductivity (21.963 mS m^−1^) of the polymeric membrane. As seen on the Nyquist diagram, it appears as a straight line with a slope of 30°, which is identified as Warburg impedance. The straight line following the semicircle, which slopes at 30°, is characteristic of a Warburg impedance, indicating diffusion-controlled processes. Typically, a slope of 45° is characteristic of semi-infinite linear diffusion (when ion diffusion within the electrolyte or polymeric membrane is not restricted); however, the 30° slope suggests finite diffusion or limited ion diffusion. This implies that diffusion through the polymeric membrane is significant but restricted, which is potentially due to the medium’s ionic conductivity or the membrane’s thickness [38,39,40,41,42]. Such finite diffusion could be related to phenomena like ion diffusion resistance or mass transport limitations (e.g., ions moving through narrow pores or channels in the membrane). As the impedance increases from 112.903 kohm to 583.870 kohm along the straight line, the impedance increase reflects the increasing resistance due to diffusion. The fact that the line extends so far indicates that the polymeric membrane is facing significant diffusion limitations, which lead to much higher overall resistance values. This behavior is typical of systems where both charge transfer resistance and mass transport (diffusion) limitations are present [38,41,43,44]. In this case, after the initial charge transfer at the membrane/electrode interface (represented by the semicircle), the system becomes dominated by the slow diffusion of ions, leading to Warburg-type behavior (represented by the 30° line). The straight line indicates that after charge transfer, the system becomes diffusion-limited, which could severely restrict the membrane’s effectiveness in high-performance electrochemical applications despite its high electrical conductivity. EIS delivers knowledge about the resistance of transport cations across polymeric membranes and could confirm the chelating reactions between polymers, TiO_2_ nanoparticles and Cd^2+^ [38,44].

## 5. Conclusions

This paper effectively demonstrated the preparation of polymeric membranes with and without TiO_2_ nanoparticles (CA-PEG-PVST0 and CA-PEG-PVS0 membranes). The studies were conducted to investigate the feasibility of a new laboratory-scale electrochemical separation system with a custom-built setup and the efficiency of the obtained polymeric membranes for treating synthetic waste-water containing cadmium. The polymeric membranes were characterized by FTIR, SEM coupled with EDS, TGA, and impedance spectroscopy. Also, the water contact angle, water retention, and porosity of the obtained polymeric membranes were determined. The results indicated that the TiO_2_ nanoparticles were successfully incorporated into the polymeric membrane matrix and improved the amorphous structure of the polymeric membrane. These nanoparticles improved the wetting properties, thermal stability and ionic conductivity. The results indicated that the values for maximum decomposition temperature, before the electrochemical separation test, were 360.2 °C for the CA-PEG-PVST0 membrane and 351.8 °C for the CA-PEG-PVS0 membrane. After the electrochemical separation test, the values were 356.7 °C for the CA-PEG-PVST1 membrane and 351.1 °C for the CA-PEG-PVS1 membrane. The values obtained for a water contact angle of 21.5°, water retention of 79.81%, and porosity of 72.83% for the CA-PEG-PVST0 membrane confirmed the effect of the inclusion of TiO_2_ nanoparticles in the polymeric membrane matrix. The ionic conductivity values, before the electrochemical separation test, were 2.610 mS m^−1^ for the CA-PEG-PVST0 membrane and 1.771 mS m^−1^ for the CA-PEG-PVS0 membrane. After the electrochemical separation test, they were 21.963 mS m^−1^ for the CA-PEG-PVST1 membrane and 1.388 mS m^−1^ for the CA-PEG-PVS1 membrane. The high ionic conductivity of the CA-PEG-PVST1 membrane can be attributed to the fact that more free mobile cadmium ions were formed and its ability to adsorb/retain cadmium ions. It can be concluded that the higher the ionic conductivity, the better the polymeric membrane performs in high-efficiency electrochemical applications. Conversely, polymeric membranes with lower ionic conductivity may find use in applications where selectivity or robustness is more critical than the speed or efficiency of ion transport. Additionally, a key finding of this study is the high efficiency of the polymeric membrane with a low amount of TiO_2_ nanoparticles for the removal of cadmium ions. This type of possible perm-selective or ion-selective polymeric membranes can also be used in the membrane-based separation technologies for reducing contaminants from water or waste-water.

## Figures and Tables

**Table 1:** Calculated results for cadmium ions after 1.5 h of electrochemical separation test (R_Cd_^2+^ (%); CE (%); SEC (kWh L^−1^); J_Cd_^2+^ (g m^−2^ h^−1^); k (m s^−1^)).

Data	Code of Polymeric Membranes	
	CA-PEG-PVST1	CA-PEG-PVS1
R_Cd_^2+^ (%)	95.53	85.29
CE (%)	11.13	8.09
SEC (kWh L^−1^)	2.03	2.49
J_Cd_^2+^ (g m^−2^ h^−1^)	1.29	1.14
k 10^6^ (m s^−1^)	2.26	1.39

**Table 2:** Water retention (WR (%)) and porosity (ε (%)) values of the obtained polymeric membranes.

Type of Polymeric Membrane	WR (%)	ε (%)
CA-PEG-PVST0	79.81	72.83
CA-PEG-PVS0	60.76	63.16

**Table 3:** The weight loss percentage, T_max_, and residue values of the polymeric membranes, before and after the electrochemical separation tests.

**Code of** **Polymeric** **Membrane**	**Temperature Range** **(°C)**			**T_max_** **(°C)**	**Residue** **(mg)**
	30–135	135–500	500–700		(700 °C/N_2_)
	**Weight loss** **(%)**				
CA-PEG-PVST0	1.90	80.40	3.62	360.2	14.08
CA-PEG-PVS0	1.79	81.98	4.52	351.8	12.05
CA-PEG-PVST1	2.01	80.04	4.88	356.7	13.07
CA-PEG-PVS1	1.89	79.72	5.48	351.1	12.91

**Table 4:** Electrical parameters of the studied polymeric membranes (bulk membrane resistance (R_b_), charge transfer resistance (R_ct_) and ionic conductivity (σ)).

Code of Polymeric Membranes	R_b_ (kohm)	R_ct_ (kohm)	R_b_ + R_ct_ (kohm)	σ (mS m^−1^)	Observations
CA-PEG-PVST0	5.405	424.324	429.729	2.610	-
CA-PEG-PVS0	8.000	400.000	408.000	1.771	-
CA-PEG-PVST1	0.645	112.258	112.903	21.963	Warburg impedance— towards 583.870 kohm
CA-PEG-PVS1	10.204	263.709	373.913	1.388	With capacitive behavior from 373.913 kohm

## Data Availability

The original contributions presented in this study are included in the article. Further inquiries can be directed to the corresponding author.

## References

[1] Szwaczko K., Kołodyńska D., Beata Podkościelna B. (2025). Functionalized TEVS-EGDMA microspheres for efficient cadmium(II) removal: Synthesis, characterization, and adsorption performance. Sep. Purif. Technol..

[2] Khan Z., Elahi A., Bukhari D.A., Rehman A. (2022). Cadmium sources, toxicity, resistance and removal by microorganisms-A potential strategy for cadmium eradication. J. Saudi Chem. Soc..

[3] WHO (2018). A Global Overview of National Regulations and Standards for Drinking Water Quality.

[4] UNEP (2010). Final Review of Scientific Information on Cadmium.

[5] Kubier A., Wilkin R.T., Pichler T. (2019). Cadmium in soils and groundwater: A review. Appl. Geochem..

[6] European Union (1998). Council Directive 98/83/EC on the Quality of Water Intended for Human Consumption.

[7] Min K.J., An H.J., Park K.Y. (2023). Cadmium-treatment efficiency and membrane fouling during electrodialysis of waste-water discharged from zinc smelting. Chemosphere.

[8] Marimuthu T., Ching Y.C., Meriam N.S.N., Gunathilake T.M.S.U., Ng C.A. (2022). Chitosan/chitin whiskers composite membranes with polyethylene, glycol for removal of cadmium ions. Desal. Water Treat..

[9] Abdel-Karim A., Gad-Allah T.A., El-Kalliny A.S., Ahmed S.I.A., Souaya E.R., Badawy M.I., Ulbricht M. (2017). Fabrication of modified polyethersulfone membranes for waste-water treatment by submerged membrane bioreactor. Sep. Purif. Technol..

[10] Behboudi A., Jafarzadeh Y., Yegani R. (2017). Polyvinyl chloride/polycarbonate blend ultrafiltration membranes for water treatment. J. Membr. Sci..

[11] Farahbakhsh J., Delnavaz M., Vatanpour V. (2017). Investigation of raw and oxidized multiwalled carbon nanotubes in fabrication of reverse osmosis polyamide membranes for improvement in desalination and antifouling properties. Desalination.

[12] Liang S., Qi G., Xiao K., Sun J., Giannelis E.P., Huang X., Elimelech M. (2014). Organic fouling behavior of superhydrophilic polyvinylidene fluoride (PVDF) ultrafiltration membranes functionalized with surface-tailored nanoparticles: Implications for organic fouling in membrane bioreactors. J. Membr. Sci..

[13] DeFriend K.A., Wiesner M.R., Barron A.R. (2003). Alumina and aluminate ultrafiltration membranes derived from alumina nanoparticles. J. Membr. Sci..

[14] Filimon A., Dobos A.M., Onofrei M.D., Serbezeanu D. (2025). Polyvinyl Alcohol-Based Membranes: A Review of Research Progress on Design and Predictive Modeling of Properties for Targeted Application. Polymers.

[15] Osman A.I., Chen Z., Elgarahy A.M., Farghali M., Mohamed I.M.A., Priya A.K., Hawash H.B., Yap P. (2024). Membrane Technology for Energy Saving: Principles, Techniques, Applications, Challenges, and Prospects. Adv. Energ. Sustain. Res..

[16] Özkök S.C., Esra Altıok E., Bunani S., Ipekçi D., Kabay N., Arda M., Yüksel M. (2024). Application of Electrodialysis Reversal Method for Concentrate Management of Reverse Osmosis Process Following MBR Treatment of Wastewater. J. Membr. Sci. Res..

[17] Khraisheh M., Elhenawy S., AlMomani F., Al-Ghouti M., Hassan M.K., Hameed B.H. (2021). Recent Progress on Nanomaterial-Based Membranes for Water Treatment. Membranes.

[18] Xiong C., Wang W., Tan F., Luo F., Chen J., Qiao X. (2015). Investigation on the efficiency and mechanism of Cd(II) and Pb(II) removal from aqueous solutions using MgO nanoparticles. J. Hazard. Mater..

[19] Ayyaru S., Dinh T.T.L., Ahn Y.-H. (2020). Enhanced antifouling performance of PVDF ultrafiltration membrane by blending zinc oxide with support of graphene oxide nanoparticle. Chemosphere.

[20] Dammak L., Fouilloux J., Bdiri M., Larchet C., Renard E., Baklouti L., Sarapulova V., Kozmai A., Pismenskaya N. (2021). A Review on Ion-Exchange Membrane Fouling during the Electrodialysis Process in the Food Industry, Part 1: Types, Effects, Characterization Methods, Fouling Mechanisms and Interactions. Membranes.

[21] El-Shamy A.S., Emara M.M., Shahba R.M.A., Shoeeb A.M. (2021). Application of electrodialysis technique in removal of some heavy metal ions from discharge waste-water in paper industry. Al Azhar Bull. Sci..

[22] Căprărescu S., Modrogan C., Purcar V., Dăncilă A.M., Orbulet O.D. (2021). Study of Polyvinyl Alcohol-SiO_2_ Nanoparticles Polymeric Membrane in Waste-water Treatment Containing Zinc Ions. Polymers.

[23] Sandu T., Chiriac A.L., Tsyntsarski B., Stoycheva I., Căprărescu S., Damian C.M., Iordache T.V., Patroi D., Marinescu V., Sârbu A. (2021). Advanced hybrid membranes for efficient nickel retention from simulated waste-water. Polym. Int..

[24] Lee G. (2011). Effects of operating parameters on the removal performance of electrodialysis for treating waste-water containing cadmium. Desal. Water Treat..

[25] Keun Ho C., Jeoung T.K. (2002). Removal of zinc ions in waste-water by electrodialysis. J. Chem. Eng..

[26] Santarosa V.E., Peretti F., Caldart V., Zoppas J., Zeni M. (2002). Study of ion-selective membranes from electrodialysis removal of industrial effluent metals II: Zn and Ni. Desalination.

[27] Sadrzadeh M., Razmi A., Mohammadi T. (2007). Separation of different ions from waste-water at various operating conditions using electrodialysis. Sep. Purif. Technol..

[28] Liu Y., Ke X., Zhu H., Chen R., Chen X., Zheng X., Jin Y., Van der Bruggen B. (2020). Treatment of raffinate generated via copper ore hydrometallurgical processing using a bipolar membrane electrodialysis system. Chem. Eng. J..

[29] Mikhaylin S., Bazinet L. (2016). Fouling on ion-exchange membranes: Classification, characterization and strategies of prevention and control. Adv. Colloid Interface Sci..

[30] Hosseini S.M., Askari M., Koranian P., Madaeni S.S., Moghadass A.R. (2004). Fabrication and electrochemical characterization of PVC based electrodialysis heterogeneous ion exchange membranes filled with Fe_3_O_4_ nanoparticles. J. Ind. Eng. Chem..

[31] Pereira V.R., Isloor A.M., Bhat U.K., Ismail A.F., Obaidd A., Fun H.-K. (2015). Preparation and performance studies of polysulfone-sulfated nano-titania (S-TiO_2_) nanofiltration membranes for dye removal. RSC Adv..

[32] Isaac J.A., Mangani L.M., Devaux D., Bouchet R. (2022). Electrochemical Impedance Spectroscopy of PEO-LATP Model Multilayers: Ionic Charge Transport and Transfer. ACS Appl. Mater. Interfaces.

[33] Nile B.K., Faris A.M., Alesary H.F., Jafar N.N.A., Ismail H.K., Abdulredha M., Al Juboury M.F., Hassan W.H., Ahmed L.M., Abid H.R. (2024). Simulation study of a practical approach to enhance cadmium removal via biological treatment by controlling the concentration of MLSS. Sci. Rep..

[34] Wibowo D., Mustapa F., Selviantori S., Idris M., Mahmud A., Maulidiyah M., Muzakkar M.Z., Umar A.A., Nurdin M. (2023). CA/PEG/chitosan membrane incorporated with TiO_2_ nanoparticles for strengthening and permselectivity membrane for reverse osmosis desalination. Environ. Nanotechnol. Monit. Manag..

[35] Peyravi M., Jahanshahi M., Rahimpour A., Javadi A., Hajavi S. (2014). Novel thin film nanocomposite membranes incorporated with functionalized TiO_2_ nanoparticles for organic solvent nanofiltration. Chem. Eng. J..

[36] Yu Z., Liu X., Zhao F., Liang X., Tian Y. (2015). Fabrication of a low-cost nano-SiO_2_/PVC composite ultrafiltration membrane and its antifouling performance. J. Appl. Polym. Sci..

[37] Damavandi F., Aroujalian A., Salimi P. (2023). TiO_2_ nanoparticle stability via polyacrylic acid-binding on the surface of polyethersulfone membrane: Long-term evaluation. J. Ind. Eng. Chem..

[38] Aparicio G.M., Vargas R.A., Bueno P.R. (2019). Protonic conductivity and thermal properties of cross-linked PVA/TiO_2_ nanocomposite polymer membranes. J. Non-Cryst. Solids.

[39] Shafiq M., Sabir A., Islam A., Khan S.M., Gull N., Hussain S.N., Butt M.T.Z. (2018). Cellulaose acetate based thin film nanocomposite reverse osmosis membrane incorporated with TiO_2_ nanoparticles for improved performance. Carbohydr. Polym..

[40] Ahmad J., Deshmukh K., Habib M., Hägg M.B. (2014). Influence of TiO_2_ Nanoparticles on the Morphological, Thermal and Solution Properties of PVA/TiO_2_ Nanocomposite Membranes. Arab. J. Sci. Eng..

[41] Sugumaran J., Ahmad A.L., Zaulkiflee N.D. (2020). Improvement of ionic conductivity of titanium dioxide incorporated PVDF-HFP/cellulose acetate electrolyte membrane. IOP Conf. Ser. Mater. Sci. Eng..

[42] Lee K.H., Chu J.C., Kim A.R., Kim H.G., Yoo D.J. (2021). Functionalized TiO_2_ mediated organic-inorganic composite membranes based on quaternized poly(arylene ether ketone) with enhanced ionic conductivity and alkaline stability for alkaline fuel cells. J. Membr. Sci..

[43] Bae B., Miyatake K., Watanabe M. (2010). Sulfonated poly(arylene ether sulfone ketone) multiblock copolymer with highly sulfonated block. Synthesis and properties. Macromolecules.

[44] Siekierka A., Nowicka J., Ostrowska M. (2023). Mechanism of selective transportation of metal ions across chelating membranes in electrodialysis. Chem. Eng. Process. Process Intensif..

